# Overexpression of chloroplast NADPH-dependent thioredoxin reductase in *Arabidopsis* enhances leaf growth and elucidates *in vivo* function of reductase and thioredoxin domains

**DOI:** 10.3389/fpls.2013.00389

**Published:** 2013-10-08

**Authors:** Jouni Toivola, Lauri Nikkanen, Käthe M. Dahlström, Tiina A. Salminen, Anna Lepistö, hb Florence Vignols, Eevi Rintamäki

**Affiliations:** ^1^Molecular Plant Biology, Department of Biochemistry, University of TurkuTurku, Finland; ^2^Structural Bioinformatics Laboratory, Department of Biosciences, Åbo Akademi UniversityTurku, Finland; ^3^Centre National de la Recherche Scientifique and Laboratoire Résistance des Plantes aux Bio-agresseurs, UMR186 IRD-University of Montpellier2-CIRAD, Institut de Recherche pour le DéveloppementMontpellier, France; ^4^Department of Biological and Environmental Sciences, University of GothenburgGothenburg, Sweden

**Keywords:** chloroplast, thioredoxins, NTRC, 3-D model, carbon metabolism, redox regulation, overexpression, biomass yield

## Abstract

Plant chloroplasts have versatile thioredoxin systems including two thioredoxin reductases and multiple types of thioredoxins. Plastid-localized NADPH-dependent thioredoxin reductase (NTRC) contains both reductase (NTRd) and thioredoxin (TRXd) domains in a single polypeptide and forms homodimers. To study the action of NTRC and NTRC domains *in vivo*, we have complemented the *ntrc* knockout line of *Arabidopsis* with the wild type and full-length *NTRC* genes, in which 2-Cys motifs either in NTRd, or in TRXd were inactivated. The *ntrc* line was also transformed either with the truncated NTRd or TRXd alone. Overexpression of wild-type NTRC promoted plant growth by increasing leaf size and biomass yield of the rosettes. Complementation of the *ntrc* line with the full-length *NTRC* gene containing an active reductase but an inactive TRXd, or vice versa, recovered wild-type chloroplast phenotype and, partly, rosette biomass production, indicating that the NTRC domains are capable of interacting with other chloroplast thioredoxin systems. Overexpression of truncated NTRd or TRXd in *ntrc *background did not restore wild-type phenotype. Modeling of the three-dimensional structure of the NTRC dimer indicates extensive interactions between the NTR domains and the TRX domains further stabilize the dimeric structure. The long linker region between the NTRd and TRXd, however, allows flexibility for the position of the TRXd in the dimer. Supplementation of the TRXd in the NTRC homodimer model by free chloroplast thioredoxins indicated that TRXf is the most likely partner to interact with NTRC. We propose that overexpression of NTRC promotes plant biomass yield both directly by stimulation of chloroplast biosynthetic and protective pathways controlled by NTRC and indirectly via free chloroplast thioredoxins. Our data indicate that overexpression of chloroplast thiol redox-regulator has a potential to increase biofuel yield in plant and algal species suitable for sustainable bioenergy production.

## INTRODUCTION

Thioredoxins (TRX) are crucial components of the regulatory redox networks in all living cells. In the reduced state, TRXs control functions of cellular proteins by reducing disulphide bridges in the redox active site of a target protein. Subsequently, the oxidized thioredoxins are reduced by thioredoxin reductases (TR). TR-dependent reduction of cellular proteins by TRXs is called a thioredoxin system. Plant chloroplasts have versatile thioredoxin systems including two reductases dependent on ferredoxin (FTR) and NADPH (NTR) as reducing power, respectively, and multiple types of TRXs (f, m, x, y, z, CDSP32; Buchanan and Balmer, 2005; Meyer et al., 2008; Jacquot et al., 2009; König et al., 2012). Plastid-localized NADPH-dependent thioredoxin reductase (NTRC) is a unique NTR enzyme constituting a thioredoxin system in a single polypeptide chain (Serrato et al., 2004; Pérez-Ruiz et al., 2006). In NTRC, a TRX module is fused to the C-terminus of a reductase domain. The protein contains two redox-active 2-Cys motifs, CAIC in its NTR domain (NTRd) and CGPC in the TRX domain (TRXd; Serrato et al., 2004), and it is suggested to function as a dimer (Pérez-Ruiz and Cejudo, 2009; Pérez-Ruiz et al., 2009; Lee et al., 2012). Characterization of knockout lines of *NTRC* (*ntrc*) has indicated that NTRC is a crucial redox-regulator of a number of plastidial processes, including biogenesis of chloroplasts, biosynthetic pathways and ROS metabolism in chloroplasts (Pérez-Ruiz et al., 2006; Stenbaek et al., 2008; Michalska et al., 2009; Lepistö et al., 2009, 2012; Pulido et al., 2010; Kirchsteiger et al., 2012; Chae et al., 2013; Richter et al., 2013). 2-Cys-peroxiredoxins and ADP -glucose pyrophosphorylase, the H_2_O_2_-detoxification enzymes and the key enzyme in starch synthesis, respectively, are the most conclusively documented target proteins of NTRC (Pérez-Ruiz et al., 2006; Michalska et al., 2009; Pulido et al., 2010).

According to the reaction mechanism model of the NTRC dimer (Pérez-Ruiz and Cejudo, 2009; Lee et al., 2012), the redox-active site of the NTRd of one subunit reduces the disulphide bridge of TRXd in the second subunit, which subsequently reacts with NTRC target proteins. *In-vitro* assays with purified NTRC and its target proteins support the model, demonstrating that the NTRd of NTRC primarily reduces its own TRXd, whereas it has a poor capability of reacting with other chloroplast TRXs (Pérez-Ruiz and Cejudo, 2009; Bohrer et al., 2012; Lee et al., 2012). However, recombinant NTRC protein forms oligomeric aggregates *in vitro* (Wulff et al., 2011) that may inhibit the interactions of NTRC with other chloroplast TRXs. To study the action of the NTRC domains *in vivo*, we have complemented the *ntrc* knockout line with a wild type *NTRC* gene and with full-length genes, in which the redox-active Cys motif in the NTRd (C217S/C220S) or in the TRXd (C454S/C457S) was inactivated. The *ntrc* line was also independently transformed either with a truncated NTRd or TRXd domain. Here we show that overexpression of the full-length NTRC with inactivated redox active Cys residues either in the NTRd or the TRXd partly complemented the *ntrc* mutant phenotype in *Arabidopsis*. The mutated NTRC proteins were capable of dimerization *in vivo*. Modeling of the three-dimensional structure of NTRC dimers indicates extensive interactions at the dimeric interface. It is also likely that the mutated NTRC acted via other chloroplast thioredoxin systems in restoring chloroplast development and the activity of metabolic pathways. We demonstrate here that thioredoxin f (TRXf) is the most prominent partner to interact with NTRC *in vivo*. Finally, overexpression of wild type NTRC promoted leaf expansion and dry weight accumulation of *Arabidopsis* rosettes, especially under increased light intensity.

## MATERIALS AND METHODS

### PLANT TRANSFORMATION AND DNA ANALYSIS

**FIGURE 1:**
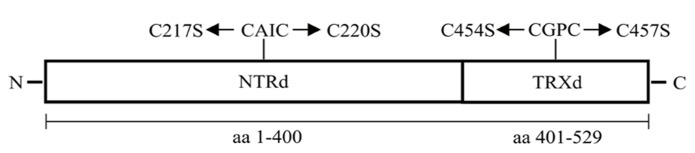
**Schematic representation of the NTRd and TRXd and the active site motifs of *Arabidopsis* NTRC.** The amino acid sequence presented in the figure includes a chloroplast transit peptide of 67 amino acids at the N-terminus. Mutations of Cys residues in the redox active site of NTRd and TRXd are indicated in the figure.

An *NTRC* coding sequence of *Arabidopsis thaliana *(At2g41680) containing a chloroplast signal sequence was used as a template in PCR to amplify a full-length *NTRC* (*OE-NTRC*), a truncated N-terminal NTRd encoding the amino acids 1–400 of NTRC (*OE-NTRd*), and a truncated TRXd encoding the amino acids 401–529 of NTRC (*OE-TRXd*; **Figure 1**; **Table 1**). A putative transit peptide of *NTRC* consisting of 67 amino acids (ChloroP-program, Emanuelsson et al., 1999) was fused into an N-terminus of a truncated TRXd. The sequences were cloned as *Nco*I/*BamH*I fragments into the pGWR8 plasmid (Rozhon et al., 2010) under control of the cauliflower mosaic virus (CaMV) 35S promoter. The calculated molecular masses of mature NTRC, NTRd and TRXd (without chloroplast transit peptide) are 50890, 35796, and 15100 Da, respectively.

**Table 1:** Forward and reverse primers for overexpression (OE) and yeast two-hybrid (Y2H) bait and prey constructions.

	Sequence	Constructs used for
**OE-primers**		
**OE.NTRC**		
Frw	5′-CTGCCATGG*ATG*GCTGCGTCTCCCAAGA-3′	OE-NTRC, OE-SAIS, OE-SGPS
Rev	5′-CGGGATCCTCATTTATTGGCCTCAATGAAT-3′	
**OE.NTRd**		
Frw	5′-CTGCCATGG***ATG***GCTGCGTCTCCCAAGA-3′	OE-NTRd
Rev	5′-CGGGATCC***TCA***AAATTCAACAAGAAGATTGTT-3′	
**SS_NTRC**		
Frw	5′-CTGCCATGG***ATG***GCTGCGTCTCCCAAGA-3′	Cloning of SS to TRXd
Rev	5′-GGAGACTCTGAGGCGGAGTCCC\underbarCCCGGG-3′	
**OE.TRXd**		
Frw	5′-TCCCCCCGGGCACCAGCCTCAAACTGAAGA-3′	OE-TRXd
Rev	5′-CGGGATCC***TCA***TTTATTGGCCTCAATGAAT-3′	
**Y2H primers**		
**Y2H.NTRC**
Frw	5′-CGCAT***ATG***GCTGCGTCTCCCAAGA-3′	pGBKT7.NTRC, pGADT7.NTRC, pGBKT7.NTRC^CAIS^, pGADT7.NTRC^CAIS^, pGBKT7.NTRC^CGPS^, pGADT7.NTRC^CGPS^, pGBKT7.NTRC^SGPS^
Rev	5′-CGGGATCC***TCA***TTTATTGGCCTCAATGAATT-3′			
**Y2H.NTRd**
Frw	5′- GGGAATTC\underbar CAT***ATG***GCCACCGCCAATTCTCCG-3′	pGBKT7.NTRd, pGADT7.NTRd, pGBKT7.NTRC^SAIS^
Rev	5′-CGGGATCC***TCA***AAATTCAACAAGAAGATTGTT-3′	
**Y2H.TRXd**		
Frw	5′-GGGAATTCCAT***ATG***CACCAGCCTCAAACTGAAGA-3′	pGBKT7.TRXd, pGADT7.TRXd, pGADT7.TRXd^SGPS^
Rev	5′-CGGGATCC***TCA***TTTATTGGCCTCAATGAATT-3′	
**Mutagenesis primers**		
**NTRC**^**C217S**^		
Frw	5′-GATAAGTGCTAGTGCTATCAG-3′	OE-SAIS, pGBKT7.SAIS
Rev	5′-CTGATAGCACTAGCACTTATC-3′	
**NTRC**^**C454S**^		
Frw	5′-TCACCAACAAGTGGCCCCA-3′	OE-SGPS, pGBKT7.NTRC^SGPS^, pGADT7.TRXd^SGPS^
Rev	5′-TGGGGCCACTTGTTGGTGA-3′	
**NTRC**^**C220S**^		
Frw	5′-GATAAGTGCTTGTGCTATCAG-3′	OE-SAIS, pGBKT7.NTRC^CAIS^, pGBKT7.NTRC^SAIS^, pGADT7.NTRC^CAIS^, pGADT7.NTRC^SAIS^
Rev	5′-CTGATAGCACAAGCACTTATC-3′	
**NTRC**^**C457S**^		
Frw	5′-ATGTGGCCCCAGTAGGACTC-3′	OE-SGPS, pGBK7.NTRC^CGPS^, pGADT7.NTRC^CGPS^, pGBKT7.NTRC^SGPS^, pGADT7.TRXd^SGPS^
Rev	5′-GAGTCCTACTGGGGCCACAT-3′	

*Restriction enzyme sites are underlined and translational start and stop codons are marked by bold italic. NTRd = NTR domain and TRXd = TRX domain without any mutations. CAIS and SAIS = Second or both redox cysteines in the NTRd of the full-length NTRC are switched to serine. CGPS and SGPS = Second or both redox cysteines in the TRXd of the full-length NTRC are switched to serine. SS = chloroplast signal sequence of NTRC. Frw = forward primer, rev = reverse primer.*

Full-length *NTRC* was used as a template to generate monocysteine NTRC mutants with a C220S mutation in the NTRd or a C457S mutation in the TRXd by using QuikChange XL Site-Directed Mutagenesis Kit (Agilent Technologies, Stratagene, Santa Clara, CA, USA; **Table 1**). These constructs were used as templates to make double cysteine mutants, C217S/C220S in the NTRd, or C454S/C457S in the TRXd (**Figure 1**), which were then named as *OE-SAIS *and *OE-SGPS *constructs, respectively. All plasmids were sequenced.

Overexpression constructs *OE-NTRC*, * OE-SAIS*, * OE-SGPS, OE-NTRd*, **and* OE-TRXd *were introduced to electrocompetent *Agrobacterium tumefaciens* strain GV3101 by standard electroporation protocol using Gene Pulser cuvettes (Bio-Rad, CA, USA). Transformed agrobacterium cells were selected by growing for two days at 29°C on LB agar plates in the presence of 20 μg/ml of rifampicin, 50 μg/ml of gentamicin sulfate, 5 μg/ml of tetracycline hydrochloride and 50 μg/ml of kanamycin. All antibiotics were purchased from Sigma-Aldrich (St. Louis, MO, USA). Transformation of *ntrc* knockout plants (SALK_096776^1^, Alonso et al., 2003; Lepistö et al., 2009) was done according to standard floral dipping procedures (Clough and Bent, 1998). Plants treated with agrobacterial suspension were grown in a growth chamber under long day conditions (16 h light/8 h dark) until seeds were collected. Selection of transformed seeds was carried out in 0.6% agar containing 0.5× Murashige and Skoog basal salt mixture (MS; Sigma-Aldrich) and 50 μg/ml of kanamycin. Resistant seedlings were transferred into soil and plants were grown in a growth chamber under long day conditions until the harvest of seeds (T2 seeds).

### MATERIALS AND GROWTH CONDITIONS

Seeds of the T2 or T3 generations of two independent transgenic lines (*OE-NTRC-18 and 22, OE-SAIS-57 and 58*, *OE-SGPS-11 and 12*, *OE-NTRd-13 and 1*4, and* OE-TRXd-15 and 27)* in *ntrc* background were used in the experiments, except in **Table 3**, in which the biomass yield of the five additional independent transgenic *OE-NTRC* lines indicated in the table was measured. Seeds were first germinated on agar plates containing 50 μg/ml kanamycin and the resistant seedlings were transferred into soil. Wild type *Arabidopsis *ecotype Columbia (WT), T-DNA insertion mutant of NTRC (*ntrc*) and transgenic lines overexpressing wild type or mutant NTRC proteins in *ntrc* background were grown on a mixture of soil and vermiculite (1:1) under 130 and 600 μmol of photons m^-2^ s^-1^ at 23°C under short day (8-h light/16-h dark) or long day (16-h light/8-h dark) conditions as indicated in figures and tables.

### DETERMINATION OF ROSETTE DRY WEIGHT, CHLOROPHYLL CONTENT, PHOTOCHEMICAL EFFICIENCY OF PHOTOSYSTEM II, AND STARCH CONTENT OF LEAVES

Leaf number of five rosettes of WT, *ntrc* and transgenic lines overexpressing wild-type or mutated NTRC was counted, the rosettes were then dried at 60°C for 24 h and weighed there-after.

Five leaf disks, 5 mm in diameter were incubated in 1 ml of 100% dimethylformamide (DMF) (Mallinckrodt Inc.) overnight at 4°C in darkness. Total content of chlorophyll and chlorophyll a/b ratio (Chl a/b) of the solutions were measured at wavelengths 646.6, 663.6 and 750 nm with Lambda 25 UV/VIS Spectrometer (Perkin Elmer, MA, USA). Chlorophyll concentrations were calculated according to Porra et al. (1989).

The photochemical efficiency of Photosystem II in intact leaves illuminated under growth light intensity for 2 h was measured with a Hansatech PEA fluorometer after a 30-min dark incubation and recorded as the ratio of variable to maximal fluorescence (Fv/Fm), where Fv is the difference between maximal fluorescence (Fm) and initial fluorescence (Fo).

For detection of starch content leaves were detached from rosettes after 4 h of illumination and incubated in DMF until chlorophyll was bleached. Plants were rinsed with water, stained with Lugol solution (5% I2 and 10% KI) in distilled water with total iodine content of 130 mg/ml for 2 min, destained with water for 1 h and photographed (Lepistö et al., 2009).

### EXTRACTION OF SOLUBLE LEAF PROTEINS, SDS-PAGE AND WESTERN BLOTTING

*Arabidopsis* leaves were frozen in liquid N_2_, and proteins were extracted in buffer containing 50 mM HEPES (Fisher Scientific, UK), 5 mM NaCl (Mallinckrodt Inc., Phillipsburg, NJ, USA), 10 mM MgCl_2_ (Sigma-Aldrich) and with and without 2 mM DTT (Lepistö et al., 2009). Soluble protein extracts were solubilized with and without mercaptoethanol at 0°C or heated at 100°C for 1 min (Laemmli, 1970). The amounts of protein indicated in the figures were loaded on a gel containing 10% (w/v) acrylamide in the separation gel. After separation in SDS-PAGE the proteins were subsequently electroblotted to a PVDF membrane (Millipore^2^; Lepistö et al., 2009). Wild type and mutated NTRC as well as the truncated TRXd of NTRC were detected by NTRC-specific antibody that was raised against the amino acids 475–488 in the TRXd (Lepistö et al., 2009). Because the anti-NTRC antibody does not recognize the truncated NTRd, its overexpression in transgenic lines was detected by anti-NTRB antibody raised against wheat NTRB enzyme (kindly provided by prof. F.J. Cejudo, Institute of Plant Biochemistry, University of Sevilla). Anti-Rubisco antibody (Agrisera), cross-reacting with the large subunit of Rubisco, was used for the detection of Rubisco content in leaf extracts. Molecular mass markers were purchased from New England Biolabs (NEB, MA, USA).

### Y2H BINARY ASSAYS

Full length and truncated *NTRC* sequences described in Section “Plant Transformation and DNA Analysis” and **Figure 1** were subcloned as DNA fragments between the NdeI(5′) and BamHI(3′) restriction sites of both pGAD.T7 and pGBK.T7 yeast two-hybrid vectors (Clontech, TAKARA BIO INC., Japan). These constructs were used as templates for site-directed mutagenesis to generate single (CAIS, CGPS) and double (SGPS) mutants. All plasmids were checked by sequencing.

Y2H experiments were conducted in the yeast reporter strain CY306 (Vignols et al., 2005), a Y2H strain designed for stabilizing redox interactions between TRXs and their target proteins (Vignols et al., 2005). CY306 strain was cultured in YNB with 0.7% yeast extract w/o amino acids (BD Difco, NJ, USA) and 2% glucose monohydrate (Mallinckrodt) supplemented with required amino acids and bases (Sigma-Aldrich), and co-transformed with different sets of pGAD and pGBK constructs carrying *NTRC* and derived sequences using lithium acetate as previously described (Vignols et al., 2005; Lepistö et al., 2013; **Table 1**). CY306 double transformants were selected as cells growing in the absence of leucine and tryptophan, and further assayed for Y2H interaction in the absence of tryptophan, leucine and histidine. Interaction between the viral genome-linked protein VPg and eukaryotic translation initiation factor eIF4G (Hébrard et al., 2010) was used as a positive control, while empty pGADT7 or pGBKT7 vectors served as negative controls in the Y2H tests.

### TRANSMISSION ELECTRON MICROSCOPY

Leaves of 6-week-old plants were collected after the dark period, fixed with 3% glutaraldehyde in 100 mM sodiumphosphate buffer, pH 7.0, placed under a vacuum for 2.5 h, thereafter fixed and stained according to Pätsikkä et al. (2002), and examined with a transmission electron microscope (JEM-1400 Plus TEM, JEOL Ltd., Tokyo 196-8558, Japan).

### STRUCTURAL MODELING OF NTRC

**FIGURE 2:**
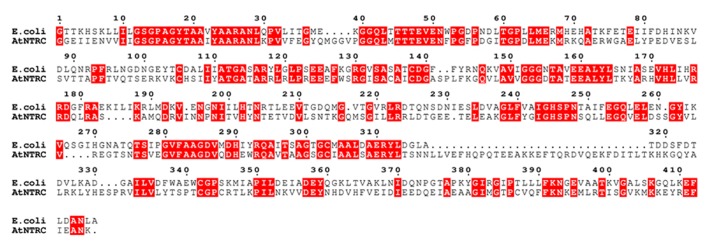
**Sequence alignment used for modeling.** The sequence alignment used for modeling shows the *E. coli* NTR and the TRX sequences aligned to the NTRC sequence. Conserved residues are shown in red boxes.

To search for related sequences and a crystal structure that could be used as a template for modeling, the *Arabidopsis* NTRC sequence was used as bait to search UniProtKB and Protein Data Bank (PDB) with the Basic Local Alignment Search Tool (BLAST) at NCBI^3^. A homology model of NTRC (residues 79–529) was constructed based on the crystal structure of the NTR–TRX complex from *Escherichia coli *(PDB code 1F6M; Lennon et al., 2000). MALIGN (Johnson et al., 1996) in the BODIL modeling environment (Lehtonen et al., 2004) was used to align NTRC with similar sequences and the *E. coli* NTR sequence, which shares 43% sequence identity to NTRC (**Figure 2**). A separate alignment was made for the TRXd of NTRC and the *E. coli* TRX sequence, together with several other known TRX structures. For modeling, all sequences except NTRC and the *E. coli* sequences were excluded from the alignments. The NTRd and the TRXd sequence alignments were then combined into one alignment, which was used for modeling (**Figure 2**). The linker region connecting the NTRd to the TRXd was determined based on sequence alignment with barley NTRC, which has an approximately 35 amino acids long linker between residues 341 and 374 (Wulff et al., 2011). *Arabidopsis* NTRC shares 81 % identity to barley NTRC, indicating a similar linker region. Based on the alignment, the *Arabidopsis* NTRC linker is located between amino acids 396 and 429, i.e., 34 amino acids long. Residues 406 to 421 of the linker were restrained to an α-helix as predicted by the JPred (Cole et al., 2008) and PSIPred (Jones, 1999) secondary structure prediction programs. A set of ten models was created with MODELLER (Sali and Blundell, 1993), and the model with the lowest value of the MODELLER objective function was analyzed and compared to the crystal structure of the *E. coli *NTR–TRX complex by superimposition with VERTAA (Johnson and Lehtonen, 2000) in BODIL. The quality of the final model was assessed with PROCHECK (Laskowski et al., 1993) and QMEAN (Benkert et al., 2009). The same was done for all ten different TRXs found in *Arabidopsis* chloroplasts, which were modeled to the NTR domain of NTRC. The APBS tool in PyMOL (Version 1.4, Schrödinger, LLC) was used to calculate electrostatic surfaces. PyMOL was also used to prepare pictures of the 3D model and ESPript (Gouet et al., 1999) for alignment pictures.

## RESULTS

### NTRC CONTENT IN TRANSGENIC OVEREXPRESSION LINES

**FIGURE 3:**
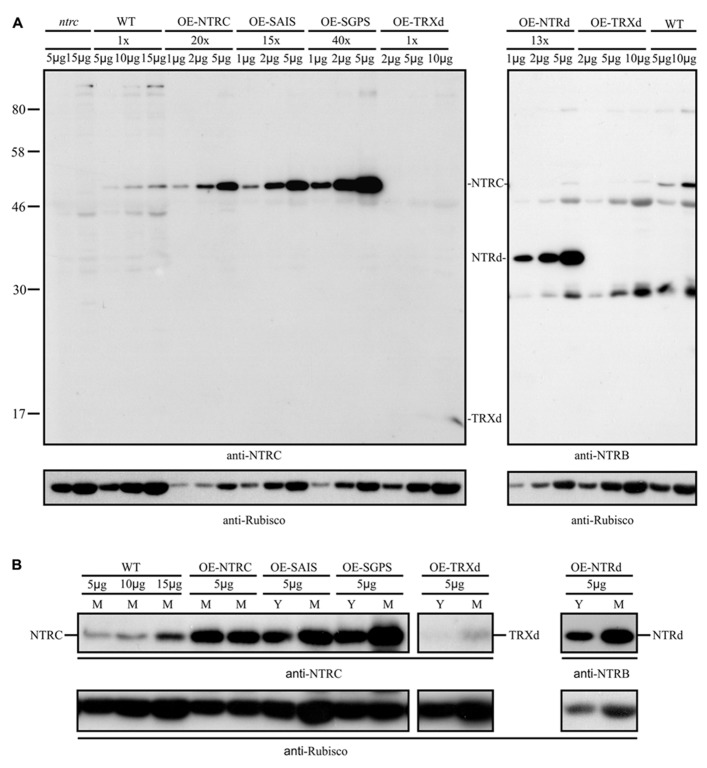
**(A)** NTRC content in the wild type (WT) and in the transgenic lines overexpressing wild-type, mutated NTRC, or truncated NTRC domains. Different amounts of leaf extracts (μg soluble protein, upper panel) were loaded on SDS-gel and the proteins were separated in SDS-PAGE before Western blotting with anti-NTRC antibody, anti-NTRB antibody, and anti-Rubisco antibody. The content of mutated NTRC and truncated NTRC domains (NTRd and TRXd) in leaf extracts of transgenic lines were normalized to Rubisco content and presented in the upper panel relative to the amount of NTRC in wild type (WT). Molecular mass standard (kDa) is presented in left side of the blot.**OE-NTRC, OE-SAIS, OE-SGPS, OE-NTRd, and OE-TRXd represent the overexpression lines of *NTRC*, *NTRC *with inactive reductase or thioredoxin active sites, and the transgenic lines overexpressing truncated NTRd or TRXd, respectively. *ntrc *is a knockout line of *NTRC*. In addition to NTRC, anti-NTRB antibody cross-reacts with *Arabidopsis* proteins of the apparent molecular masses of 80, 46, and 30 kDa. **(B)** Increase in accumulation of NTRC protein during maturation of transgenic leaves overexpressing mutated NTRC, or truncated NTRC domains. Amounts of leaf extracts (μg soluble protein, upper panel) were loaded on SDS-gel and the proteins were separated by SDS-PAGE before Western blotting with anti-NTRC antibody, anti-NTRB antibody, and anti-Rubisco antibody. Y and M; soluble proteins were extracted from yellowish and green leaves, respectively.

To study the function of NTRC domains *in vivo*, we complemented the *ntrc* line with a wild type *NTRC* gene (*OE-NTRC *lines) and with full-length genes, in which the redox-active 2-Cys motifs of the NTRd (*OE-SAIS *lines) or TRXd (*OE-SGPS *lines) were inactivated by site-directed mutations (**Figure 1**). The *ntrc* line was also independently transformed either with a truncated NTRd (*OE-NTRd *lines) or TRXd (*OE-TRXd* lines). The expression levels of transgenic genes were detected by immunoblotting with both an NTRC-specific antibody and with an antibody raised against the NTRB enzyme from wheat. In comparison to WT, the content of NTRC protein was ten to forty times higher in the leaves of transgenic lines except in the *OE-TRXd* line, in which the accumulation of truncated TRXd equalled the content of NTRC in WT leaves (**Figure 3**). Despite high overexpression of transgenic genes no degradation products of NTRC were detected in leaf extracts indicating that the mutated NTRC protein was stable in transgenic lines. The phenotype complementation of transgenic lines in *ntrc* background was not caused by the leaking of the original T-DNA insertion in endogenous *NTRC* gene, since no full-length NTRC protein was detected in the transgenic lines expressing truncated NTRC domains (**Figure 3**).

### OVEREXPRESSION OF NTRC IN *ARABIDOPSIS*

**FIGURE 4:**
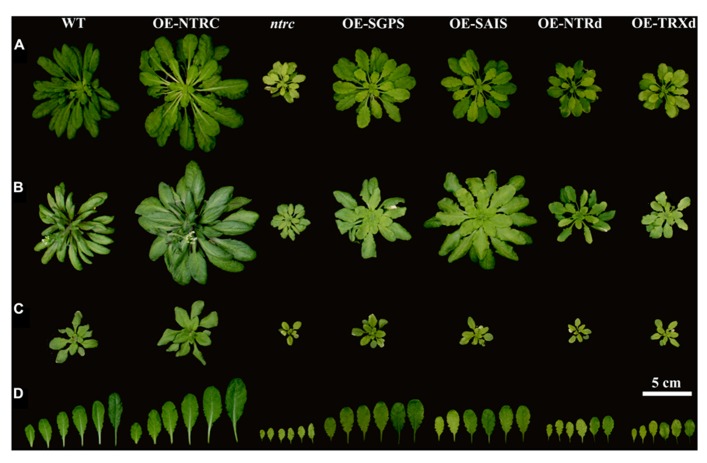
**Phenotypes of transgenic lines overexpressing wild-type, mutated NTRC, or truncated NTRC domains.** Wilt type (WT), knockout line of *NTRC* (*ntrc*), and the overexpression lines of mutated and truncated *NTRC* are grown under short day conditions **(A)** at 130 μmol of photons m^-2^ s^-1^, or **(B)** at 600 μmol of photons m^-2^ s^-1^, and **(C)** under long day conditions at 600 μmol of photons m^-2^ s^-1^. **(D)** Largest rosette leaves of plants presented in **(A)**. For abbreviations see the legend of the **Figure 3**.

**FIGURE 5:**
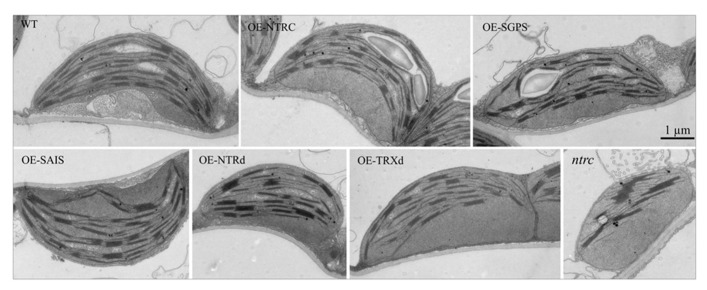
**Chloroplast ultrastructure in transgenic lines overexpressing wild-type, mutated NTRC or truncated NTRC domains.** Plants were grown at 600 μmol of photons m^-2^ s^-1 ^under short day conditions for 6 weeks before sampling of the leaves. Scale bar is 1 μm. For abbreviations see the legend of the **Figure 3**.

Complementation of the *ntrc* line with the wild-type *NTRC* gene fully recovered the green phenotype of seedlings, the wild-type growth rate of rosettes and the ultrastructure of chloroplasts (**Figures 4 and 5**; **Table 2**). Yellowish leaves typical for *ntrc* mutant lines were not detected at any developmental stage of leaves. In comparison to WT, the rosette dry weight of OE-NTRC lines was about 40% higher in 7-weeks-old *OE-NTRC* lines grown at 130 μmol photons m^-2^ s^-1^ (**Table 2**). Overexpression of *NTRC* did not increase the number of leaves in these plants, but the size of the fully expanded *OE-NTRC* leaves was substantially larger (**Figure 4**). Increase in light intensity further stimulated the biomass yield of transgenic *OE-NTRC* plants in comparison to WT (**Table 2**). For validation of positive effect of *NTRC* overexpression on biomass yield we measured the dry weight of six independent transgenic *OE-NTRC* lines grown under 600 μmol photons m^-2^ s^-1^ for 6 weeks (**Table 3**). The data showed that overexpression of *NTRC* generally increased the biomass yield of rosettes albeit the extent of growth stimulation varied between lines.

**Table 2:** Leaf number, chlorophyll content and dry weight of transgenic rosettes overexpressing wild-type, mutated NTRC, or truncated NTRC domains.

Line	Chl a + b (μg cm^-2^ ± SE) (% of WT)	Chl a/b ± SE	Dry weight (mg ± SE) (% of WT)	Leaf number ± SE
***SD 130 μmol m*^-2^ s^-1^**		
**WT**	26.3 ± 1.2 (100)	3.13 ± 0.08	197 ± 18 (100)	38 ± 1
**OE-NTRC**	19.9 ± 1.8 (76)*	3.13 ± 0.08	280 ± 46 (142)	39 ± 1
***ntrc***	3.0 ± 0.5 (11)***	2.61 ± 0.09**	24 ± 5 (12)***	22 ± 1***
**OE-SGPS**				
Young	6.0 ± 0.2 (23)***	2.78 ± 0*	108 ± 12 (55)**	37 ± 1
Mature	16.4 ± 1.6 (63)**	3.09 ± 0.04		
**OE-SAIS**				
Young	8.3 ± 0.6 (32)***	2.98 ± 0.07	102 ± 7 (52)**	34 ± 1*
Mature	14.1 ± 0.7 (54)***	3.18 ± 0.07		
**OE-NTRd**				
Young	5.9 ± 0.5 (22)***	2.85 ± 0.10	51 ± 3 (26)***	30 ± 1***
Mature	10.9 ± 0.7 (41)***	3.13 ± 0.04		
**OE-TRXd**	ND	ND	56 ± 2 (28)***	28 ± 1***
***SD 600 μmol m*^-2^s^-^1**				
**WT**	13.5 ± 0.4 (100)	3.90 ± 0.04	334 ± 13 (100)	ND
**OE-NTRC**	14.9 ± 0.2 (110)*	3.90 ± 0.08	877 ± 57 (263)***	ND
***ntrc***	7.5 ± 0.5 (55)***	3.35 ± 0.09***	33 ± 4 (10)***	ND
**OE-SGPS**				
Young	7.9 ± 0.5 (58)***	3.93 ± 0.11	176 ± 16 (53)**	ND
Mature	14.5 ± 1.1 (107)	4.01 ± 0.10		
**OE-SAIS**				
Young	8.6 ± 0.8 (63)***	3.75 ± 0.08	354 ± 47 (106)	ND
Mature	14.9 ± 0.7 (110)	4.09 ± 0.05*		
**OE-NTRd**				
Young	9.0 ± 1.0 (67)**	3.93 ± 0.06	112 ± 18 (34)***	ND
Mature	16.7 ± 1.1 (124)*	4.02 ± 0.06*		
**OE-TRXd**				
Young	7.7 ± 0.4 (57)***	3.86 ± 0.10	45 ± 5 (13)***	ND
Mature	10.4 ± 0.7 (77)**	4.08 ± 0.08		

Plants were grown under short day conditions (SD) for 7 weeks in the light intensity indicated in the table. Leaf number, chlorophyll content (Chl a+b), chlorophyll a/b ratio (Chl a/b) and rosette dry weight were measured as described in Section “Materials and Methods.” Each value is the mean ± SE of four to five independent determinations. Chlorophyll content of transgenic lines overexpressing mutated NTRC was measured from both young yellowish and green (mature) leaves. ND, not determined. *P, **P, ***P < 0.05, 0.01, 0.001 (student’s *t*-test), respectively, compared with WT plants. For the other abbreviations, see the legend for **Figure 3**.

**Table 3:** Dry weight and leaf number of transgenic rosettes overexpressing wild-type, mutated NTRC, or truncated NTRC domains.

Line	Dry weight mg ± SE (% of WT)	Leaf number±SE
***LD 600 μmol m*^-2^s^-1^**		
WT	129 ± 17 (100)	13 ± 1
OE-NTRC	257 ± 17 (200)***	17 ± 1**
*ntrc*	29 ± 1 (23)***	10 ± 1* OE-SGPS	89 ± 7 (69)*	12 ± 1
OE-SAIS	67 ± 5 (52)**	12 ± 1
OE-NTRd	28 ± 2 (22)***	9 ± 1**
OE-TRXd	34 ± 4 (26)***	11 ± 1*
***SD 600 μmol m*^-2^s^-1^**		
WT	235 ± 13 (100)	33 ± 2
OE-NTRC 4	286 ± 14 (122)*	36 ± 1
OE-NTRC 6	316 ± 21 (135)*	35 ± 2
OE-NTRC 8	334 ± 7 (142)***	35 ± 1
OE-NTRC 9	379 ± 55 (162)*	38 ± 1
OE-NTRC 10	446 ± 38 (190)**	41 ± 3
OE-NTRC 18	336 ± 33 (143)*	39 ± 3

Plants were grown at 600 μmol of photons m^-2^ s^-1^ under long day conditions (LD) for 3 weeks or under short day conditions (SD) for 6 weeks. Leaf number and rosette dry weight were measured as described in Section “Materials and Methods.” The numbers in OE-NTRC lines represents independent transgenic lines. Each value is the mean ± SE of four to five independent determinations. ND, not determined. *P, **P, ***P < 0.05, 0.01, 0.001 (student’s *t*-test), respectively, compared with WT plants. For the other abbreviations, see the legend of **Figure 3**.

**FIGURE 6:**
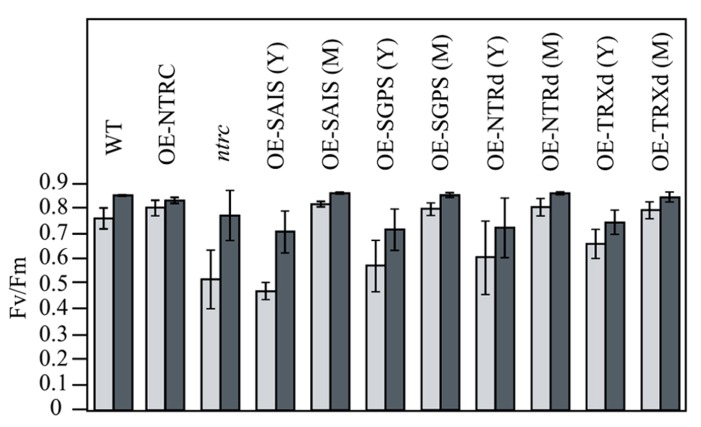
**Recovery of photochemical efficiency of Photosystem II in transgenic lines overexpressing wild-type, mutated NTRC, or truncated NTRC domains.** Plants were grown under short day condition at 130 μmol of photons m^-2^ s^-1^ (light gray bars) or at 600 μmol of photons m^-2^ s^-1 ^(dark gray bars) for 6 weeks and the photochemical efficiency of Photosystem II was measured after illumination for 2 h at growth light intensity. Each value is the mean ± SE (bars) of five independent determinations. Y and M; the photochemical efficiency of Photosystem II was measured both for young yellowish (Y) and green (M) leaves of the transgenic lines. For other abbreviations see the legend of the **Figure 3**.

**FIGURE 7:**
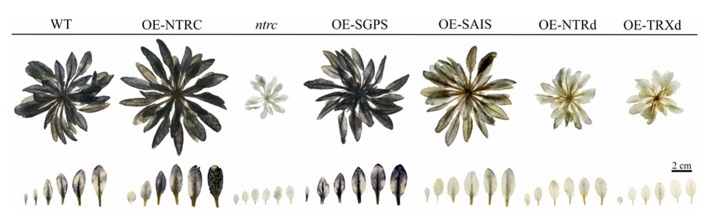
**Accumulation of starch in transgenic lines overexpressing wild-type, mutated NTRC, or truncated NTRC domains.** Seven-weeks-old rosettes were grown at 600 μmol of photons m^-2^ s^-1^ under short day condition and five rosettes were harvested after illumination for four hours. For starch staining rosettes were treated with Lugol solution as described in Section “Materials and Methods” and photographed. Blue color indicates the amount of starch in leaves. For abbreviations, see the legend of the **Figure 3**.

Chlorophyll content of *OE-NTRC* leaves in plants grown at 130 μmol photons m^-2^ s^-1^ was about 25% lower than in WT leaves, whereas slightly higher chlorophyll content was detected in plants grown at 600 μmol photons m^-2^ s^-1^ (**Table 2**). Increase in light intensity particularly reduced the amount of chlorophyll per WT leaf area. Overexpression of NTRC did not change the chlorophyll a/b ratio in leaves. Complementation of the *ntrc* line with wild type *NTRC* also fully recovered the photochemical efficiency of Photosystem II, which was significantly reduced in *ntrc *lines at growth light intensity (**Figure 6**; Lepistö et al., 2009). Overexpression of *NTRC *also**increased the accumulation of starch in illuminated leaves (**Figure 7**).

The *ntrc* lines show most severe growth defects under short day conditions that promote the vegetative growth of *Arabidopsis* (Pérez-Ruiz et al., 2006; Lepistö et al., 2009). Therefore we studied, whether the overexpression of NTRC also promotes *Arabidopsis* growth under long day conditions that induce early flowering in *Arabidopsis*. Similarly to short day conditions, three weeks-old *OE-NTRC* plants grown at 600 μmol photons m^-2^ s^-1 ^showed substantial increase in rosette dry weight and a higher number and larger size of leaves (**Figure 4**; **Table 3**). We conclude that overexpression of NTRC significantly stimulates leaf expansion in *Arabidopsis* and the increase in light intensity promotes the gain of leaf biomass in *OE-NTRC* transgenic lines.

### PHENOTYPES OF PLANTS OVEREXPRESSING MUTATED NTRC GENES AND TRUNCATED NTRC DOMAINS

Complementation of the *ntrc* line with the full-length *NTRC* gene containing an active reductase but inactive thioredoxin domain (*OE-SGPS*), or vice versa (*OE-SAIS*), substantially accelerated the growth of transgenic plants in comparison to the *ntrc* line (**Figure 4**; **Tables 2 and 3**). In plants grown at 130 μmol photons m^-2^ s^-1^ for 7 weeks the rosette dry weight of the *ntrc* line was less than 15% of the wild type, whereas transgenic plants with an active reductase or Trx catalytic site produced about 50% of the wild type dry mass. Overexpression of the *OE-SGPS* and *OE-SAIS* mutant genes in *ntrc *background partially recovered the chloroplast ultrastructure (**Figure 5**) as well as increased the chlorophyll content (**Table 2**) and restored the photosynthetic function of mature leaves (**Figure 6**). The young leaves, particularly in the *OE-SAIS* transgenic lines, were yellowish in color but became green during expansion and aging of leaves (**Figure 4**; **Table 2**). The greening of the leaves correlated with the accumulation of transgenic NTRC protein in the leaves (**Figure 3B**), suggesting that the mutated NTRC protein was less competent in the complementation of the knockout of the endogenous NTRC, but high accumulation of mutated NTRC proteins promoted greening and recovery of leaf photosynthetic activity. The slow greening rate of young leaves reduced the growth rate of the *OE-SGPS* and the *OE-SAIS* transgenic lines, resulting in substantial reduction of rosette dry mass in comparison to WT under all growth light conditions tested. The *OE-SAIS* transgenic lines also accumulated less starch in light than the *OE-NTRC *or the* OE-SGPS* lines (**Figure 7**). The *OE-SGPS* and *OE-SAIS* transgenic lines demonstrate that the full-length NTRC with an active reductase but inactive TRXd, or vice versa, is capable of regulating chloroplast processes to some extent.

Overexpression of truncated forms of NTRC containing either NTRd or TRXd sequences in *ntrc* background complemented only poorly the mutant phenotype of *ntrc* (**Figures 3 and 4**; **Tables 2 and 3**). The content of truncated NTRd in the *OE-NTRd* transgenic lines was high and no proteolytic degradation products were detected by Western blotting (**Figure 3**), indicating that the lack of phenotypic complementation was due to low catalytic activity of the NTRd in the absence of full-chain NTRC polypeptide. The truncated TRXd is likely unstable, since high accumulation of the TRXd was not detected in *OE-TRXd* lines. An increase in the content of truncated NTRC proteins during leaf expansion induced slow greening of the leaves with increased amount of chlorophyll per leaf area and resulted in the recovery of the photosynthetic activity of old green leaves (**Figures 3–6**; **Table 2**). However, due to the inefficient complementation of chloroplast function by the truncated forms of NTRC, the growth rate of these transgenic plants was only slightly accelerated in comparison to the *ntrc* line. The *OE-NTRd *and *OE-TRXd* lines demonstrate that the NTRd and TRXd alone can only poorly form a catalytically competent enzyme capable of interacting with plastidial proteins.

### DIMERIC STRUCTURE OF NTRC

The phenotype of transgenic lines overexpressing mutated full-length NTRC proteins indicates that the intact catalytic site of NTRC remains active in chloroplasts, albeit less efficient in regulation of chloroplast proteins than the WT enzyme. Furthermore, removing either the NTRd or TRXd from the full-length NTRC abolished the activity of the remaining domain. Eukaryotic NTRs are strictly homodimeric proteins (Hirt et al., 2002), which led us to ask if the reduced catalytic activity of NTRC in transgenic lines overexpressing the mutated NTRC or truncated NTRC domains is due to an inability to form homodimers *in vivo*.

**FIGURE 8:**
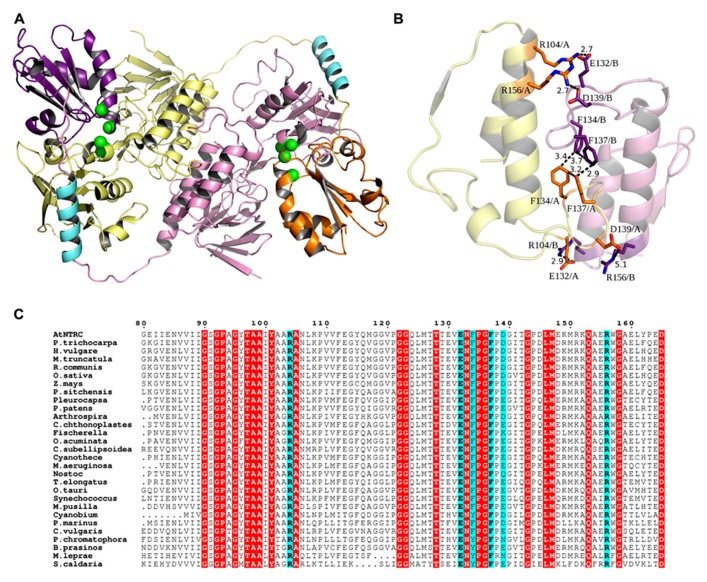
**(A)** Dimer model of NTRC. In the dimer, the TRX domain (orange and purple) of one subunit binds to the NTR domain (yellow and pink) of the other subunit and, thus, tightens the inter-monomeric interactions. Within one subunit, a long linker region that starts from the C-terminus of the NTR domain and forms a short α-helix (cyan) connects to the N-terminus of the TRX domain, suggesting possible flexibility for the position of the TRX domain. Active site cysteines are shown as green spheres. **(B)** Dimer interactions. In addition to the arrangement of subunits and the linker-mediated interactions, five possible salt bridges are formed between the FAD binding domains. Four of these are shown as sticks in the picture (orange and purple), and three of these are at an optimal distance (~2.7 Å) from each other. Additionally, four phenylalanines in the middle of the interface interact with each other (pi-pi stacking) in a square. Altogether, these special interactions can account for the experimentally observed strong dimeric interactions. **(C)** NTRC like sequences. A part of the multiple sequence alignment of NTRC like sequences from *Arabidopsis thaliana* (UniProtKB O22229), *Populus trichocarpa* (UniProtKB B9H9S9), *Hordeum vulgare* (UniProtKB B0FXK2), *Medicago truncatula* (UniProtKB A6XJ27), *Ricinus communis* (UniProtKB B9SU44), *Oryza sativa Japonica Group* (UniProtKB Q70G58), *Zea mays* (GenBank DAA63960.1), *Picea sitchensis* (UniProtKB B8LPW6), *Pleurocapsa* sp.* PCC 7327* (UniProtKB K9T492), *Physcomitrella patens *subsp.* patens* (UniProtKB A9U311), *Arthrospira *sp.* PCC 8005* (UniProtKB H1WE73), *Coleofasciculus chthonoplastes PCC 7420* (UniProtKB B4VQ87), *Fischerella *sp.* JSC-11* (UniProtKB G6FX47), *Oscillatoria acuminata PCC 6304* (UniProtKB K9TN60), *Coccomyxa subellipsoidea C-169* (UniProtKB I0Z4C2), *Cyanothece *sp.* PCC 7822* (UniProtKB E0U9J8), *Microcystis aeruginosa PCC 7941* (UniProtKB I4GKJ6), *Nostoc *sp.* PCC 7120 *(UniProtKB Q8YYV6), *Thermosynechococcus elongatus BP-1* (UniProtKB Q8DHM2), *Ostreococcus tauri* (UniProtKB Q00SZ2), *Synechococcus *sp.* PCC 6312* (UniProtKB K9RZN2), *Micromonas pusilla CCMP1545* (UniProtKB C1N9L2), *Cyanobium *sp.* PCC 7001* (UniProtKB B5INL4), *Prochlorococcus marinus *subsp.* marinus str. CCMP1375* (UniProtKB Q7VB53), *Chlorella vulgaris* (UniProtKB B9ZYY5), *Paulinella chromatophora* (UniProtKB B1X3G8), *Bathycoccus prasinos* (UniProtKB K8EJ46), *Mycobacterium leprae TN* (UniProtKB P46843) and *Spirochaeta caldaria DSM 7334* (UniProtKB F8F2K7) show that the residues forming salt bridges of optimal length (cyan) are mostly conserved between species. Red boxes indicate other conserved residues.

The 3D model of the NTRC homodimer is presented in **Figure 8A**. The overall flavin reducing structure of the model shows the fold required for the reaction mechanism of the NTRC dimer (Pérez-Ruiz and Cejudo, 2009; Lee et al., 2012). The linker that connects the C-terminus of the NTRd and the N-terminus of the TRXd is ~34 amino acids long and is likely to form a short α-helix. In the dimer, the TRXd of one subunit interacts with the NTRd of the other subunit. In addition to this arrangement, the FAD binding domains also strengthen the dimer interactions. The inter-monomeric interactions are formed by eleven hydrogen bonds and five possible salt bridges, three of which are at an optimal distance from each other (~2.7 Å; **Figure 8B**). Additionally, there are four phenylalanines interacting with each other (pi-pi stacking) in a square.

**FIGURE 9:**
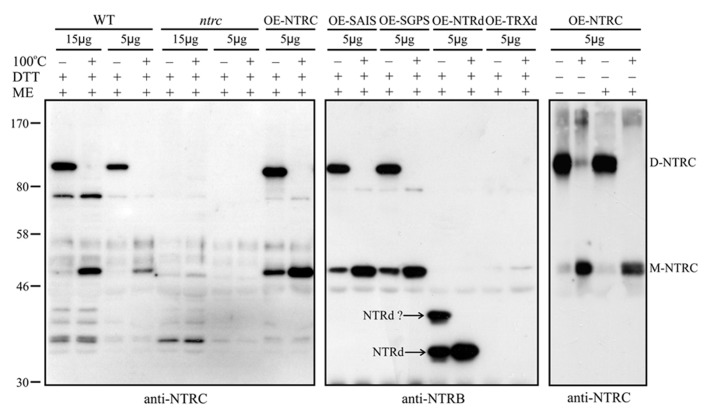
**Heat-induced monomerization of NTRC homodimers.** Proteins were extracted from leaves in the presence (+) or absence (-) of DTT and solubilized in SDS-buffer with (+) or without (-) mercaptoethanol (ME; Laemmli, 1970) on ice (-) or heated at 100°C for 1 min (+). Amounts of leaf extracts (μg soluble protein, upper panel) were loaded on SDS-gel and the proteins were separated in SDS-PAGE before Western blotting with anti-NTRC antibody or anti-NTRB antibody. D-NTRC and M-NTRC, dimeric and monomeric NTRC proteins, respectively. NTRd?, truncated NTRd with anomalous movement in SDS-gel. For other abbreviations, see the legend for **Figure 3**.

To analyze the oligomeric form of NTRC *in vivo*, soluble proteins were extracted from WT, *ntrc* and transgenic lines overexpressing mutated and truncated NTRC proteins in the presence or absence of thiol reducing chemical. The protein samples were solubilized with SDS either with or without mercaptoethanol and kept on ice or heated as described in Section “Materials and Methods” before the separation of proteins in SDS-PAGE. While the NTRC band of heated samples corresponded to the monomeric full-length NTRC with an apparent molecular mass of 50 kDa, the molecular mass of the major NTRC band in unheated samples was approximately 100 kDa, representing the homodimer of NTRC (**Figure 9**). Dimeric NTRC was detected both with NTRC-specific and anti-NTRB-antibodies and it was totally absent in protein extract from *ntrc* lines. The dimeric form of NTRC structure was very stable because it was present in the samples treated with SDS and mercaptoethanol and kept on ice before SDS-PAGE, and it did not monomerize until heating of the protein extract. The heat-induced monomerization of polypeptides took place also without addition of thiol-reducing compounds, although extraction of proteins without DTT caused slight smearing of NTRC polypeptides in the SDS gel (**Figure 9**). The monomerization of NTRC can be explained by the absence of disulphide bridges at the dimer interface of NTRC (**Figure 8B**). Therefore, the inter-monomeric aromatic interactions, together with the salt bridges, and the interlocking arrangement of the TRXds, account for the strong dimeric interactions we observed. This is further supported by the fact that the salt bridges of optimal length are conserved in the alignment of NTRC like sequences (**Figure 8C**). F137 is also conserved in most sequences, but F134 is sometimes substituted by tryptophan or tyrosine, both of which retain the aromatic stacking property. K, Q or N occasionally replaces D139, but these substitutions retain the hydrogen bonding capacity at this position.

The Western blots demonstrated that WT and the mutated full-length NTRC proteins formed homodimers *in vivo* (**Figure 9**). The unheated protein samples extracted from the *OE-NTRd* lines also gave two bands, which cross-reacted with the anti-NTRB antibody. The apparent molecular mass of the band with lower mobility was less than 50 kDa, which is much lower than the estimated molecular mass of a homodimeric NTRd (72 kDa). Heating of the protein extract removed the upper band and concomitantly increased the intensity of the band of monomeric NTRd (36 kDa), indicating that both bands consisted of truncated NTR polypeptides (**Figure 9**). The anomalous migration of NTRd in SDS-gel and the high content of monomeric form in unheated protein sample suggest that the truncated NTRd has reduced capability of forming dimers *in vivo*. No homodimer of TRXd was detected in transgenic lines overexpressing truncated TRXd.

**FIGURE 10:**
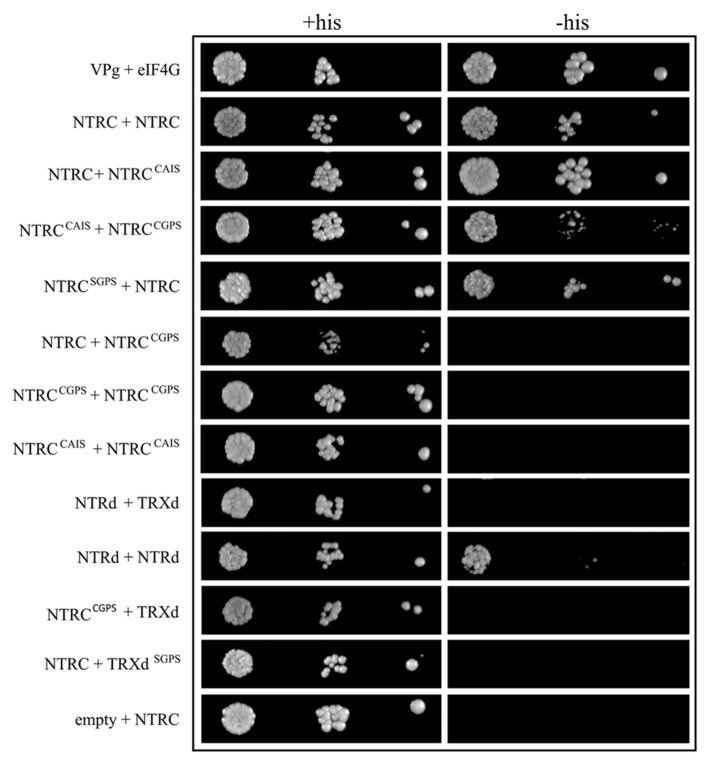
**Interactions of the wild-type, mutated, and truncated NTRC in Y2H tests.** Yeast CY306 cells were transformed with NTRC, truncated NTRd or TRXd, and with mutated NTRC constructs with both or the second Cys of the redox active site of NTRd (CAIS construct), or TRXd (SGPS and CGPS constructs, respectively) replaced by Ser. The cells were grown into stationary phase and adjusted to an OD_600_ of 0.05, 0.005 and 0.0005 before spotting onto plates containing histidine (+His) or without histidine (-His). Yeast cells were photographed after four days growth at 28°C. VPg + elF4G and empty vector was used as positive and negative controls, respectively (see Materials and Methods).

The self-interaction of the wild-type NTRC proteins was confirmed by binary Y2H test (**Figure 10**). Neither NTRC nor NTR polypeptide without TRXd (NTRd) interacted with a truncated wild-type (TRXd) or with mutated TRX polypeptide (TRXd^SGPS^) in the Y2H test, whereas truncated NTRd did interact with itself. To study the effect of altered redox state of NTRC active sites on the interactions of polypeptides, the second Cys in the catalytic site either of the NTRd (NTRC^CAIS^) or TRXd (NTRC^CGPS^) was mutated to Ser. The CAIS-form of the NTRd active site reacts with the disulphide of a target TRX resulting in a stable mixed disulphide between the NTRd active site and TRX. The CGPS-form of the TRXd active site is permanently reduced and thus cannot react with the NTRd active site of NTRC. The self-interaction of the NTRC proteins was detected, when wild-type NTRC was tested with NTRC^CAIS^ or NTRC^SGPS^, and when NTRC^CAIS^ was tested with NTRC^CGPS^, whereas neither NTRC^CAIS^ nor NTRC^CGPS^ was capable of self-interaction. Neither NTRC with a monothiol in the TRXd active site (NTRC^CGPS^) interacted with a truncated TRX polypeptide (TRXd) in the Y2H test.

## DISCUSSION

Thioredoxin reductases are homodimeric enzymes that catalyze the NADPH-dependent reduction of cellular thioredoxins (Hirt et al., 2002; Bernal-Bayard et al., 2012). We report here that the full-length NTRC polypeptides extracted from *Arabidopsis* leaves in the presence or absence of DTT (**Figure 9**) exist as homodimers that are resistant to detergent treatment without heating, suggesting that the dimeric structure of chloroplast NTRC is stable without any disulphide bridges between the monomers (**Figure 8B**). This conclusion is further confirmed by the observation that the mutation of both Cys residues either in the NTRd or in the TRXd active site does not abolish the formation of NTRC homodimers (**Figure 9**; Pérez-Ruiz et al., 2009). The high conservation rate of the aromatic amino acids and those involved in salt bridges in the inter-monomeric interface of the NTRC domains (**Figure 8B**), suggests that these residues are important for forming stable homodimers. The interlocked arrangement of the NTRd and TRXd together with the NTR-TRX linker region further stabilizes the homodimeric architecture of *Arabidopsis* NTRC. This conclusion is supported by the observation that no homodimers were present in transgenic lines overexpressing truncated NTRd (**Figure 9**), although the Y2H test indicated that the NTRd could interact with itself (**Figure 10**). Furthermore, the linker region may facilitate the correct folding of NTRd, since an anomalous form of truncated NTRd was detected in SDS-PAGE (**Figure 9**). The position of the linker is still uncertain, since it could fold on the side of the NADPH-binding domain as in our models (**Figure 8A**) or into the groove in front of the protein as Lee et al. (2012) suggested. The linker placement could also differ depending on the state and conformation of the protein, which is determined by the rotation of the NADPH-binding domain. Similarly to the crystal structure of AhpF (PDB code 1HYU; Wood et al., 2001), which has a short α-helical linker between the NTRd and TRXd, the relatively long linker (~34 amino acids) in *Arabidopsis* NTRC is likely to form an α-helix as predicted.

The binary Y2H tests showed that the TRX active site of NTRC has to be intact or both Cys residues mutated to Ser in order to support interactions of NTRC polypeptides, since a monothiol form of the TRXd active site repulsed the self-interactions of NTRC polypeptides in Y2H tests (**Figure 10**; NTRC + NTRC^CGPS^, NTRC^CGPS^ + NTRC^CGPS^). The repulsion was not detected when the interaction of NTRC^CAIS^ was tested with NTRC^CGPS^ (**Figure 10**). In this case two thiols instead of three exist in the contacting region of mutated TRXd and NTRd in a dimer that mimics the conditions in wild-type NTRC during reduction of TRXd by NTRd. Furthermore, NTRC^CAIS^ failed to interact with itself, which may be caused by artificial aggregation of NTRC^CAIS^ proteins. NTRC^CAIS^ makes a stable mixed-disulphide with the active site of the TRXd in the second NTRC polypeptide, which may be linked with the third polypeptide by disulphide formation resulting in an oligomerization of polypeptides. Such an aggregated molecule is likely incapable of activating the reporter gene system in yeast cells. Poor complementation of *ntrc* phenotype in *OE-NTRd *and *OE-TRXd* lines further support our conclusion that only full-length NTRC is capable of forming a stable dimeric, functional NTRC enzyme.

NADPH-dependent thioredoxin reductase has been shown to form aggregates *in vitro*, although contradictory conclusions have been drawn on the generation and activity of oligomeric forms of NTRC. Pérez-Ruiz et al. (2009) demonstrated that rice recombinant NTRC protein or NTRC extracted from *Arabidopsis* chloroplasts form oligomeric aggregates that dissociate into the dimeric form in the presence of NADPH, whereas NADPH did not influence the dissociation of barley NTRC aggregates *in vitro* (Wulff et al., 2011). Chae et al. (2013) reported a heat-shock induced aggregation of NTRC and that the oligomeric NTRC acts as a chaperone preventing stress-induced aggregation of chloroplast proteins. They also demonstrated that only homodimeric NTRC has a disulphide reductase activity, while Wulff et al. (2011) reported that neither reductase nor thioredoxin activity was affected by oligomerization of NTRC. These studies indicate that NTRC has a strong tendency to form oligomeric aggregates but it is still technically difficult to prove, whether oligomeric NTRC is the dominant form of the enzyme *in vivo*. Pérez-Ruiz et al. (2009) demonstrated that stromal fraction of chloroplasts contained oligomeric NTRC aggregates with different masses that mostly disaggregated into dimers and monomers in the presence of DTT. We also detected slight smearing of NTRC polypeptides in the SDS-gel, if leaf proteins were extracted in DTT-free buffers (**Figure 9**). Furthermore, it has been shown that heat-shock-induced oligomerization of NTRC depended on the active site cysteines *in vitro* (Chae et al., 2013). These observations suggest that oxidation of thiols in NTRC redox active sites may induce oligomerization during extraction or purification of the NTRC protein.

Overexpression of the full-length NTRC with an inactivated NTRd or TRXd active site in *ntrc* background partially complemented the mutant phenotype of *Arabidopsis* rosettes. Both mutated NTRC constructs promoted growth and recovery of photosynthetic function of transgenic lines. NTRC controls chloroplast biogenesis (Lepistö et al., 2012) and regulates the activity of chlorophyll biosynthesis enzymes (Richter et al., 2013). Greening of the *OE-SAIS *and *OE-SGPS *transgenic leaves suggests that also the mutated NTRC is to some extent capable of promoting chloroplast biogenesis. However, greening of the *OE-SAIS *lines was delayed (**Figure 4**) and these lines accumulated less starch in light than *OE-SGPS *lines (**Figure 7**), indicating that mutated NTRC with an active NTRd was more efficient in complementing the thiol redox network in chloroplasts than mutated NTRC with an active TRXd. Reduction of free chloroplast TRXs by the NTRd of NTRC would explain the partial recovery of wild-type phenotype of *OE-SGPS* lines. The 3-D model of the NTRC dimer (**Figure 8A**) showed that the TRXd is connected to the NTRd by a long linker region that allows some flexibility for the position of the TRXd. Recently, Bernal-Bayard et al. (2012) suggested that a conformational change takes place in the NTRC dimer after reduction of TRXd active site that allows the reaction between TRXd and its target protein. This structural change also exposes the NTR active site (Bernal-Bayard et al., 2012, see also the location of 2-Cys motifs of NTRC in **Figure 8A**), which may promote the interaction between NTRd of NTRC and free thioredoxins. The Y2H test failed to show any interactions of NTRC or NTRd with TRXd (**Figure 10**). However, Y2H assay might not be suitable to test interactions between dimeric NTRC and truncated TRXd, since the bulky transcription factor domain fused to the N-terminus of NTRd may interfere with the interaction (**Figure 8A**).

**FIGURE 11:**
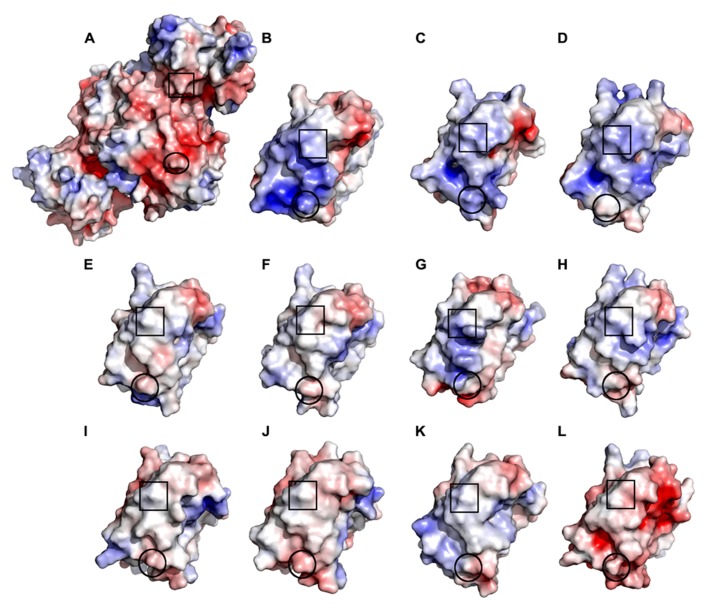
**Electrostatic surface of *Arabidopsis* NTRd and TRXd of NTRC and the free chloroplast TRXs. (A)** In the flavin reducing conformation, the NTRd of *Arabidopsis* NTRC has a negative surface charge (red) at the TRXd interaction site. **(B) **The corresponding site on the TRXd of NTRC is strongly positively charged (blue), facilitating electrostatic interactions with the oppositely charged surface of the NTRC domain. Ten free TRXs are found in *Arabidopsis* chloroplasts: TRXf1 **(C)**, TRXf2 **(D)**, TRXm1 **(E)**, TRXm2 **(F)**, TRXm3 **(G)**, TRXm4 **(H)**, TRXy1 **(I)**, TRXy2 **(J)**, TRXx **(K)** and TRXz **(L)**. Of these, TRXf1, TRXf2 and TRXm3 share a positively charged patch at the same site as TRXd of NTRC. TRXf1 and TRXf2 have only one amino acid difference in the interaction surface and a similar overall shape, indicating that these could compensate for a non-functional TRXd of NTRC. The active site cysteines are marked by boxes, while Y162 on the NTRd and position 466 on the different Trx forms are circulated. Surface charges were calculated with the APBS tool in PyMOL and the color ranged from -7 to 7.

To determine whether free chloroplast TRXs are capable of compensating for a non-functioning TRXd in the transgenic *OE-SGPS* lines, the TRXd of NTRC was supplemented by the 10 different chloroplast TRXs (TRXf1, TRXf2, TRXm1, TRXm2, TRXm3, TRXm4, TRXy1, TRXy2, TRXx, and TRXz) in the 3-D model of NTRC (**Figure 11**). Compared to the dimeric NTRC model (**Figure 8**), NTRd with TRXf1, TRXf2 and TRXm1 have only one major difference in the interacting amino acids. This residue is K466 (A-chain) in the TRXd of NTRC, which interacts with Y162 (B-chain) of the NTRd. In the other free TRX models, K466 is replaced by alanine (TRXf1, TRXx), glutamate (TRXf2, TRXm1, TRXm3, TRXy1, TRXy2), aspartate (TRXm2), glutamine (TRXm4), or methionine (TRXz). None of these is able to interact with Y162, due to their shorter length and different properties compared to K466. The models with the TRXd of NTRC replaced by TRXm2, TRXm3, TRXm4, TRXy1, TRXy2, TRXx, and TRXz have at least two additional major differences in the interacting amino acids. Electrostatic surface calculations show that the TRX contact surface on the NTRd of NTRC is strongly negatively charged, and correspondingly, the interaction site on the TRXd of NTRC is strongly positively charged (**Figure 11**). Both TRXf isoforms are similar to the TRXd of NTRC in terms of charge distribution, while the shape of TRXf1 is more similar to the TRXd than that of TRXf2. Therefore, the TRXf isoforms are the most likely candidates for supplementing the inactive TRXd of NTRC. An *in-vitro* test has shown that both NADPH-dependent thiol reductases (NTRC and NTRA) can donate electrons toTRXf1, although the reduction of TRXf was less efficient than the reduction of TRXh3, which is a natural substrate of NTRA (Bohrer et al., 2012). In these *in-vitro* assays, however, the concentration of TRXf1 was 500 times higher than the concentration of NTR enzymes (Bohrer et al., 2012), whereas the content of NTRC protein in the transgenic lines used in this article was ten to forty times higher than in WT line (**Figure 3**). Thereby the high concentration of NTRC-SGPS protein in stroma may facilitate the reduction of TRXf by NTRC that consequently mediates the redox regulation of chloroplast proteins in *OE-SGPS* lines.

Of the other chloroplast thioredoxins, TRXm1 and TRXm2 have a more neutral surface, while TRXm3 has a positively charged patch on the contact area (**Figure 11**). TRXm4, on the other hand, is weakly positively charged, while TRXy1 has a neutral surface and TRXy2 is weakly negatively charged. Hence, these are not likely to compensate for a non-functional TRXd in NTRC. TRXx and TRXz have a positively charged surface and a negatively charged surface, respectively. The negative charge on the surface of TRXz probably prevents it from interacting with the negatively charged surface on the NTRd of NTRC. TRXx, on the other hand, has a very different overall surface shape compared to the TRXd of NTRC, which makes an interaction unlikely.

**FIGURE 12:**
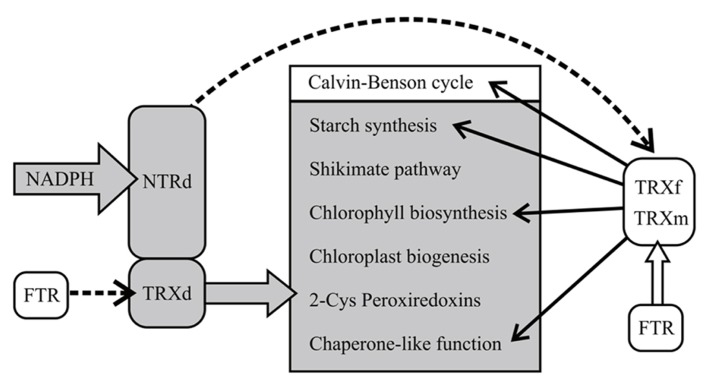
**The hypothetical model of NTRC function in transgenic lines overexpressing wild-type and mutated NTRC in chloroplasts.** Overexpression of wild-type NTRC in *Arabidopsis* stimulates chloroplast metabolism both by direct reduction of NTRC target proteins by thioredoxin domain (TRXd) of NTRC and indirectly via TRXf function. The mutated NTRC with an intact reductase domain (NTRd) regulates chloroplast metabolism via free chloroplast thioredoxins, preferably TRXf. Ferredoxin-dependent thioredoxin reductase (FTR) may reduce the TRXd in the mutated enzymes with an inactive NTRd. The dashed lines indicate an inefficient reduction of the thioredoxin by the thioredoxin reductase.

The recovery of chloroplast development and function in *OE-SAIS* lines demonstrates a presence of a reducing system in chloroplasts, likely FTR, that can donate electrons to the oxidized TRXd of NTRC in the absence of an active NTRd (**Figure 12**). The slightly delayed development and low accumulation of starch in *OE-SAIS* lines may be due to inefficient reduction of the TRXd of NTRC by FTR.

Recently, it has been proposed that the oligomeric NTRC, TRXf and TRXm have foldase (assists protein folding) and holdase (buffers proteins against aggregation) chaperone activities that are independent of the disulphide reductase activity of these proteins (Sanz-Barrio et al., 2012; Chae et al., 2013). Thus the complementation of the mutant phenotype in transgenic lines overexpressing mutated NTRC can be due to the chaperone functions of the oligomeric NTRC and be independent of disulphide reductase activity. Some experimental observations, however, speak against the phenotype of the *OE-SGPS* and *OE-SGPS* lines being largely explained by NTRC chaperone activities. Like discussed in the previous chapter, the proportion of NTRC existing as oligomers *in vivo *is not known. Furthermore, mutation in the redox active cysteines in NTRC likely reduces probability to form oligomers since heat-shock-induced oligomerization of NTRC depended on the active site cysteines *in vitro *(Chae et al., 2013). Neither chaperone activities explain why the complementation of the *ntrc* line with an *OE-SAIS* gene differs from that with an *OE-SGPS* gene (**Figures 4 and 7**). We conclude that chaperone activities of thioredoxins may only partly explain the complementation of *ntrc* line with mutated NTRC constructs.

Overexpression of wild-type *NTRC* in *ntrc* background fully recovered the wild-type structure and function of *Arabidopsis* rosettes. Moreover, high content of NTRC in leaves promoted dry mass production of rosettes, expansion of leaves, and accumulation of starch in light. Stimulation of growth was pronounced by increased growth light intensity. It has been estimated that the concentration of TRXf, TRXm and NTRC is in the range of 0.01–0.1 μM in the stroma (Peltier et al., 2006; König et al., 2012). *OE-NTRC* lines accumulated about twenty times more NTRC in leaves in comparison to WT *Arabidopsis*. Accordingly, the NTRC content in the overexpressing lines was in the range of 1 μM, which is still lower or equals to the estimated concentrations of NTRC-target enzymes in chloroplasts (enzymes in tetrapyrrole and starch synthesis, 2-Cys peroxiredoxins, Peltier et al., 2006). Interestingly, overexpression of TRXf, but not of TRXm increased biomass yield and specific leaf weight and highly stimulated the accumulation of starch and sugars in tobacco leaves (Sanz-Barrio et al., 2013). Like in the* OE-NTRC* lines of *Arabidopsis* the extent of growth stimulation in tobacco plants depended on light intensity. The overexpression of either TRXf or NTRC did not change the steady state rate of photosynthesis in tobacco (Sanz-Barrio et al., 2013) or the chlorophyll content per leaf area in *Arabidopsis* (**Table 2**). Thus the extra sugars in overexpression lines of *NTRC* and *TRXf* are likely used to expand the total leaf area (number and leaf size) per plant, which eventually increases the total photosynthesis per plant and promotes growth. Overexpression lines of *TRXf* and *NTRC* clearly prove that the plant benefits from an increase in thiol reducing systems in chloroplasts.

Specificity of TRXs to their target proteins has been under debate since the discovery of numerous TRX-types in chloroplasts (see Capitani et al., 2000; Buchanan and Balmer, 2005; Meyer et al., 2008). The observation that overexpression of TRXf, but not of TRXm (Sanz-Barrio et al., 2013), increased the biomass yield and sugar content of leaves corroborates the concept that TRXf and TRXm have different target proteins in chloroplasts. It also rules out the hypothesis that growth stimulation of transgenic lines overexpressing thioredoxins is mainly due to the chaperone activities of thioredoxins, since both TRXf and TRXm were reported to function as chaperones in tobacco plants (Sanz-Barrio et al., 2012). We demonstrate here that the electrostatic surface of the TRXd of NTRC resembles best the charges in TRXf (**Figure 11**), suggesting that NTRC and TRXf may have overlapping targets in chloroplasts. Indeed, both NTRC and TRXf have been demonstrated to activate ADP glucose pyrophosphorylase, the key enzyme of starch synthesis (Michalska et al., 2009; Thormählen et al., 2013) and the enzymes in chlorophyll biosynthesis pathway (Ikegami et al., 2007; Stenbaek et al., 2008; Richter et al., 2013). However, the growth stimulation in transgenic lines overexpressing NTRC or TRXf is hardly due to the activation of a single metabolic reaction in chloroplast. For example, redox activation of AGPase was not changed in tobacco plants overexpressing TRXf (Sanz-Barrio et al., 2013) and *Arabidopsis* transgenic lines expressing permanently reduced AGPase had an excess-starch phenotype but no stimulation of growth was reported (Hädrich et al., 2011), suggesting that stimulation of starch synthesis alone does not explain the enhanced growth of transgenic lines overexpressing NTRC or TRXf. Thereby, we propose that the increase in biomass yield is due to general promotion of chloroplast development and broader stimulation of carbon metabolism (**Figure 12**). Stimulation of growth by overexpressing TRXf and NTRC is likely based on the control of multiple biosynthetic and protective pathways in chloroplasts. Besides starch and chlorophyll biosynthesis, TRXf is a key regulator for thioredoxin-dependent enzymes in the Calvin-Benson cycle (Buchanan and Balmer, 2005; Meyer et al., 2008). Knockout of NTRC induces production of plastids with anomalous ultrastructure (Lepistö et al., 2012) suggesting that NTRC is essential for the correct biogenesis of chloroplasts. Moreover, NTRC regulates the synthesis of aromatic amino acids (Lepistö et al., 2009; Lepistö, 2011), which are precursors for the biosynthesis pathways of auxin, plant flavonoids and phenolics (Tzin and Galili, 2010). It has also been reported to protect against oxidative stress in chloroplasts (Pérez-Ruiz et al., 2006) and to increase heat-tolerance of plants (Chae et al., 2013), which may facilitate the growth of the *NTRC* overexpression line in high light. Therefore, the overexpression of NTRC may stimulate the biomass yield of plants by overall stimulation of chloroplast biosynthetic reactions, biogenesis of chloroplasts, activation state of the enzymes in the Calvin-Benson cycle via TRXf, the synthesis of aromatic amino acids and the compounds derived from them (including auxin, Lepistö et al., 2009) and finally by protecting against stresses induced by high light intensity (**Figure 12**). The chaperone-like function of chloroplast TRXs (Sanz-Barrio et al., 2012; Chae et al., 2013) may further promote metabolic homeostasis in the chloroplast.

We and Sanz-Barrio et al. (2013) have shown that overexpression of a chloroplast-localized regulatory protein stimulates biomass yield without harmful side effects on plant growth and welfare under controlled growth conditions. If the overexpression of a thiol redox-regulator turns out to permanently stimulate biomass production and the accumulation of primary carbon compounds in plants grown under various conditions, it has a potential to increase biofuel yield in plant and algal species suitable for sustainable bioenergy production.

## Conflict of Interest Statement

The authors declare that the research was conducted in the absence of any commercial or financial relationships that could be construed as a potential conflict of interest.

## Footnotes

1

2

3
